# Prevalence and determinants of utilizing skilled birth attendance during home delivery of pregnant women in India: Evidence from the Indian Demographic and Health Survey 2015–16

**DOI:** 10.1371/journal.pone.0295389

**Published:** 2024-03-07

**Authors:** Md. Akhtarul Islam, Mst. Tanmin Nahar, Tanjim Siddiquee, Afrina Sultana Toma, Farhana Hoque, Md. Zobayer Hossain

**Affiliations:** 1 Statistics Discipline, Science, Engineering & Technology School, Khulna University, Khulna, Bangladesh; 2 Development Studies Discipline, Social Science School, Khulna University, Khulna, Bangladesh; ESIC Medical College & PGIMSR, INDIA

## Abstract

**Background:**

Utilization of skilled birth attendance during home delivery of pregnant women is proven to reduce complications during and after childbirth. Though the utilization of skilled birth attendance (SBA) during home delivery has increased significantly in recent times, the rate of utilizing skilled birth attendance is still low in several regions across India. The objective of this study is to analyze the prevalence and to identify the determinants of the utilization of skilled birth attendance during home delivery of pregnant women in India.

**Methods:**

To conduct this study, data and information from the Indian Demographic and Health Survey 2015–16 have been utilized. The sample size for this study is a weighted sample of 41,171 women. The sample consisted of women who had given a live birth in the three years preceding the survey. For women with more than one child, only the first live birth was considered. The binary logistic regression model and the log-binary logistic regression analysis have been applied as the adjusted odds ratios (AORs) with 95% confidence intervals for identifying the determinants of home-based skilled birth attendance during delivery. That allows us to select the most appropriate model for our study objective by ensuring that the determinants of skilled birth attendance for home delivery are accurately assessed based on the characteristics of the data.

**Results:**

The analyses show that only 18.8% of women had utilized skilled birth attendance during delivery. Women residing in urban areas are more likely to utilize skilled birth attendance during home delivery (AOR: 1.14; 95% CI: 1.08–1.20). Women having higher education levels are associated with increased use of SBA during home delivery (AOR: 1.15; 95% CI: 1.04–1.28). Exposure to media is associated with increased utilization of SBA (AOR: 1.17; 95% CI: 1.11–1.23). Overweight women are also more likely to avail the SBA during home delivery (AOR: 1.11; 95% CI: 1.03–1.19). Women belonging to affluent households have higher odds of utilizing skilled birth attendance (AOR: 1.41; 95% CI: 1.33–1.49). Having 3+ tetanus injections is associated with the utilization of SBA (AOR: 1.56; 95% CI: 1.43–1.69). Women having 4+ antenatal care visits were more likely to utilize SBA (AOR: 1.81; 95% CI: 1.71–1.92). Women belonging to the Hindu religion were 1.12 times more likely to utilize SBA (AOR: 1.12; 95% CI: 1.07–1.18). Women with 1 to 3 birth orders were 1.40 times more likely to utilize skilled birth attendance during home delivery (AOR: 1.40; 95% CI: 1.30–1.51).

**Conclusion:**

The percentage of women utilizing skilled birth attendance during home delivery is still very low which is a matter of serious concern. Several factors have been found to be associated with the utilization of SBA during home delivery in India. As skilled birth attendance has significant positive health outcomes for pregnant women and newborns, efforts to increase the rate of SBA utilization during home delivery should be undertaken.

## Introduction

A skilled birth attendant (SBA) is a healthcare professional who provides essential and emergency healthcare services to women and their newborns during pregnancy, childbirth and the postpartum period. Delivery attended by skilled professionals is known to contribute to a better pregnancy and childbirth outcome as well as early detection and management of complications during the antenatal, delivery, and postnatal period [1]. In 2015, an estimated 3,03,000 women died from pregnancy-related causes worldwide [2]. Despite the Sustainable Development Goals (SDGs) target of reducing the global maternal mortality ratio to less than 70 per 100,000 live births by 2030, countries in WHO (World Health Organization) regions such as Sub-Saharan Africa and South Asia will have a high maternal mortality ratio (MMR) [3, 4]. Within the South Asian countries (region), India has the highest MMR [3]. Although the maternal mortality rate has decreased significantly since 1997, the rate is still high in rural and tribal areas (Meh et al., 2021) [5]. The maternal mortality rate (MMR) in India has declined from the previous three years which has been observed to be most significant in the Empowered Action Group (EAG) states and Assam (also known as low-performing states) where the number went down from 188 to 175 per 100,000 live births. The decline has been from 77 to 72 among the Southern states (known as high-performing states) [6, 7]. This decrease is strongly associated with increased utilization of essential health care and quality of care services including antenatal care, institutional delivery and skilled birth attendance (SBA). Previous research has demonstrated that unskilled birth attendance and home delivery are associated with high infant and maternal mortality and morbidity [2, 8–10]. Therefore, skilled attendants are deemed to contribute positively to the reduction of maternal and newborn mortality and morbidity [2].

However, pregnancy-related deaths among Indian women continue to be unacceptably high [6]. Despite significant progress, disparities in MMR remain widespread and prolonged across regions and socioeconomic groups in India [3, 11, 12]. Low coverage of essential maternity care services including antenatal care (ANC), SBA and postnatal care (PNC) significantly impacts mother and newborn survival. It puts them at risk [2, 13]. SBA-assisted childbirth can significantly reduce the risk of maternal and neonatal deaths caused by prematurity, intrapartum or postpartum complications [10, 14, 15]. Increasing institutional deliveries can help reduce maternal and neonatal mortality [16–18].

In three of the six WHO regions, the percentage of births attended by skilled personnel exceeds 90%. However, in certain regions where skilled birth attendance (SBA) is lacking include Africa where the figure remains less than 50% followed by South Asia [2]. SBA has increased significantly in India; for example, between 1992–93 and 2005–06, skilled birth-assisted deliveries increased by 13 percentage points (from 36 to 49 percent) and then by 81 percent in 2015–16 [12, 19]. Nonetheless, understanding regional disparities in skilled birth attendance is required as the case of India demonstrates that there are large gaps in maternity care service availability across India particularly in rural areas. There are differences in the use of SBA for deliveries between rural and urban areas with 90 and 78 percent respectively [12].

Furthermore, socioeconomic determinants are always crucial in broadening the gaps in availing SBA- assisted delivery among women in India. For example, whereas only 64% of women in the lowest income quintiles had an SBA for delivery, 96 percent of women in the highest income quintiles had availed the service in India. Ensuring safe and well-prepared childbirth requires the presence of a skilled birth attendant. In maternal and child health, it is also crucial to take into account the age of a woman at delivery as it can influence the risk of complications [20–25]. Use of SBA also varies across social groups and as per the mothers’ educational statuses [12, 26]. Furthermore, inequalities in obtaining SBA within the state and between states or regions are widespread in India. Limited research in India has investigated women’s preference towards home deliveries between public and private health facilities [27]. Several research studies have investigated the factors that influence the utilization of healthcare facilities for childbirth [28, 29]. On occasions, economic status can significantly influence the choices related to the location of childbirth than mere accessibility particularly when deciding between private and public healthcare facilities [30]. The utilization of private healthcare services is often regarded as an indicator of affluence and social standing [28]. On the other hand, public healthcare facilities serve as the primary source of cost-effective delivery facilities for India’s underprivileged populations but people may opt for home-deliveries instead [31]. Overall, most of the studies on accessing delivery services are conducted on institutional skilled birth attendance. There is a dearth of literature identifying factors affecting SBA at home which constitutes a significant literature gap. This information is critical for different stakeholders working to improve maternal and child health in India and other developing countries to make informed decisions. This study, therefore, intends to explore what factors affect home-based skilled birth attendance among Indian women.

## Materials and methods

### Sampling design and data sources

This study was conducted using the Indian Demographic and Health Survey 2015–16 data also regarded as the National Family Health Survey-4 (NFHS-4). This survey was conducted under the Ministry of Health and Family Welfare (MoHFW), Government of India. The International Institute for Population Sciences (IIPS) in Mumbai acts as the nodal agency for all the surveys conducted by the MoHFW. The 2015–16 National Family Health Survey’s primary goal was to collect vital information on health and family welfare and information on emergent difficulties in India. A two-stage stratified clustered sampling technique was used in this survey [12].

The Indian Demographic and Health Survey 2015–16 was conducted using four types of questionnaires (Household, Woman’s, Man’s, and Biomarker Questionnaire). In this study, we used data from the woman’s questionnaire. This questionnaire was based on 17 local languages administered by the Computer Assisted Personal Interviewing (CAPI) adjusted to India’s circumstances and requirements. During this survey, all eligible 15 to 49 aged women were asked questions regarding their background characteristics (for instance, age, education, religion, caste or tribe, and media exposure), reproductive history, hysterectomy prevalence, menstrual hygiene, knowledge of usage and sources of family planning methods, antenatal, delivery, postnatal and newborn care, husband’s background, fertility preferences, empowerment of women etc. [12].

In our study, we only measured the case of the first live birth in a woman’s life. A weighted sample of 41,171 women who had given a live birth in the three years preceding the survey was taken into account. Only the first live birth was considered for women who had more than one live birth.

### Outcome variable

The outcome variable for this study was whether a Skilled Birth Attendant was present at the woman’s first live birth if the delivery took place in their home. In our analysis, we considered doctors, Auxiliary Nurse Midwives (ANM)/Nurses/Midwives/Lady Health Visitors (LHV), Midwives and other health personnel as skilled birth attendants [32]. Other birth attendants like Dai (Traditional Birth Attendant) and friends/relatives were considered unskilled birth attendants. The skilled birth attendant variable is a binary response with ‘skilled provider’ coded as 1 and ‘unskilled provider’ as 0.

### Explanatory variables

For this study, the explanatory variables include sociodemographic factors such as type of place, educational level, media exposure, body mass index (BMI), tetanus injection receiving status, number of antenatal care visits, age at first birth (years), wealth index, religion and birth order. type of place was defined as two categories: ‘rural’ and ‘urban.’ Respondent’s educational level was categorized as no education, primary, secondary and higher. Media exposure was defined as women who had not watched TV or listened to the radio, or read a newspaper/magazine at least once a week and were categorized as ‘no exposure.’ In contrast, others were grouped as ‘having exposure.’ Body Mass Index was defined as BMI <18.5 as underweight, BMI between 18.5–24.9 as normal and BMI >24.9 as overweight. The Number of Tetanus injections received before birth was re-coded into None, 1, 2 and 3+. During pregnancy, the number of antenatal care visits was categorized as ‘no visits,’ ‘1 to 3 visits’ and ‘4+ visits’. Age at first birth was categorized as ≤18 years, 19–23 years and 24+ years. The wealth index was recategorized using ‘poorest’ and ‘poorer’ as ‘poor’; ‘richest’ and ‘richer’ as ‘rich’ while the middle wealth category remained the same. The respondent’s religion was re-coded into Muslim, Hindu and others. Respondent’s birth order, in this study, was re-coded into one, two, three, four and five or more.

### Statistical analyses

In this study, we first conducted a univariate analysis to observe the frequency of selected background characteristics of the women in India. Using cross-tabulation and chi-square test, the bivariate analysis shows the relationship between the outcome and explanatory variables. The Binary logistic regression (BLR) model was presented as adjusted odds ratios (AORs) with 95% confidence intervals for identifying the determinants of home-based skilled birth attendance during delivery [33]. Then, Log-binary logistic regression (LBLR) model analysis was conducted as adjusted odds ratios (AORs) (including 95% confidence intervals) for identifying the determinants of home-based skilled birth attendance. It allows for a more comprehensive analysis of the determinants of home-based skilled birth attendance. Finally, the appropriate model selection was done to decide which statistical model best approximates the reality given the set of data and minimizes loss of information. The following goodness of fit tests were used in this study for model selection: (1) Akaike information criterion (AIC) and (2) Bayesian information criterion (BIC). When the log-likelihood value is small, it indicates the model is a worse fit. After comparing BLR and LBLR models, the model with the most negligible AIC value is the best. BIC is similar to AIC. The goal of BIC is to find the best model for prediction using the highest posterior probability while the purpose of AIC is to identify the model that most plausibly generates the data. Both AIC and BIC can be used to identify whether the models are nested. It has a high potential of selecting the best model as it is independent of the order in which the models are computed. Data analyses of this study were performed through STATA version 14.0 for Windows and sampling data were weighted using the Stata Survey command.

## Results

In this study, we included 41,171 women for our analysis. As shown in Table 1, only 18.8% of women were attended by home-based skilled attendants during delivery at home. The majority of the women (87.0%) lived in rural areas and nearly two-thirds were Hindu women. About one-third of the women (29.8%) reported their education level as ‘secondary.’ More than half of the women (53.8%) had media exposure and nearly 30% of them were in the underweight group. Moreover, a high percentage of the women (73.5%) were from low-income families. More than half of the women (58.2%) received tetanus injections two times in this study. Similarly, 39.8% reported having one to three antenatal care visits before their deliveries. Most of the women (26.9%) gave birth to two children and most of the women included in this study mainly gave their first birth during 19 to 23 years of age (54.2%).

**Table 1:** Univariate analysis of the selected background characteristics of the women in India.

Variables		Frequency	Percentage (%)
**Type of place**			
	Rural	35801	87.0
	Urban	5370	13.0
**Highest educational level**			
	No education	20656	50.2
	Primary	7450	18.1
	Secondary	12270	29.8
	Higher	795	1.9
**Media exposure**			
	No	19022	46.2
	Yes	22149	53.8
**Body Mass Index**			
	Underweight	11115	27.0
	Normal	26668	64.8
	Overweight	3388	8.2
**Tetanus injection received**			
	None	8007	19.4
	1	4216	10.2
	2	23951	58.2
	3+	4997	12.1
**Antenatal care visits**			
	No visits	15962	38.8
	1–3 visits	16391	39.8
	4+ visits	8818	21.4
**Age at first birth (years)**			
	≤18	12453	30.2
	19–23	22325	54.2
	24+	6393	15.5
**Skilled birth attendants**			
	Unskilled	33428	81.2
	Skilled	7743	18.8
**Wealth index**			
	Poor	30325	73.5
	Middle	6307	15.3
	Rich	4614	11.2
**Religion**			
	Muslim	8107	19.7
	Hindu	25576	62.1
	Others	7488	18.2
**Birth order**			
	Five or more	7929	19.3
	Four	6160	15.0
	Third	9242	22.4
	Second	11087	26.9
	One	6753	16.4

Bivariate analysis (Table 2) shows significant association (p<0.001) between the utilization of home-based skilled birth attendants during delivery and the type of place, educational level, religion, media exposure, body mass index, wealth index, tetanus injection receiving status, age at first birth, number of antenatal care visits and birth order. The utilization of unskilled delivery attendants was higher among the rural woman, uneducated, women belonging to poor families and among the Hindu women. Also, there was a higher rate of using skilled birth attendants among women with one to three antenatal care visits.

**Table 2:** Bivariate analysis of different characteristics according to the use of home-based skilled birth attendance.

Variables		Skilled Birth Attendants		P-value
		Unskilled N (%)	Skilled N (%)	
**Type of place**				
	Rural	29503(71.7%)	6298(15.3%)	<0.001
	Urban	3925(9.5%)	1445(3.5%)	
**Highest educational level**				
	No education	17517(42.5%)	3139(7.6%)	<0.001
	Primary	6128(14.9%)	1322(3.2%)	
	Secondary	9260(22.5%)	3010(7.3%)	
	Higher	523(1.3%)	272(0.7%)	
**Media exposure**				
	No	16363(39.7%)	2659(6.5%)	<0.001
	Yes	17065(41.4%)	5084(12.3%)	
**Body Mass Index**				
	Underweight	9132(22.2%)	1983(4.8%)	<0.001
	Normal	21715(52.7%)	4953(12.0%)	
	Overweight	2581(6.3%)	807(2.0%)	
**Tetanus injection received**				
	None	7169(17.4%)	838(2.0%)	<0.001
	1	3484(8.5%)	732(1.8%)	
	2	18974(46.1%)	4977(12.1%)	
	3+	3801(9.2%)	1196(2.9%)	
**Antenatal care visits**				
	No visits	14043(34.1%)	1919(4.7%)	<0.001
	1–3 visits	13220(32.1%)	3171(7.7%)	
	4+ visits	6165(15.0%)	2653(6.4%)	
**Age at first birth**				
	≤18	10309(25.0%)	2144(5.2%)	<0.001
	19–23	17994(43.7%)	4331(10.5%)	
	24+	5125(12.4%)	1268(3.1%)	
**Wealth index**				
	Poor	25509(62.0%)	4741(11.5%)	<0.001
	Middle	4820(11.7%)	1487(3.6%)	
	Rich	3099(7.5%)	1515(3.7%)	
**Religion**				
	Muslim	6657(16.2%)	1450(3.5%)	<0.001
	Hindu	20446(49.7%)	5130(12.5%)	
	Others	6325(15.4%)	1163(2.8%)	
**Birth order**				
	Five or more	6952(16.9%)	977(2.4%)	<0.001
	Four	5184(12.6%)	976(2.4%)	
	Third	7510(18.2%)	1732(4.2%)	
	Second	8597(20.9%)	2490(6.0%)	
	One	5185(12.6%)	1568(3.8%)	

The Binary Logistic Regression shows that type of place, educational level, religion, media exposure, body mass index, wealth index, tetanus injection receiving status, number of antenatal care visits and birth order have highly significant association with the utilization of home-based skilled birth attendance (Table 3).

**Table 3:** Logistic regression estimates showing adjusted odds ratios (AORs) with 95% confidence intervals for determinants of using home-based skilled birth attendance.

Predictors		B	AOR	P-value	95% Confidence Interval	
					Lower	Upper
**Type of place**						
	Rural ^rc^					
	Urban	0.18	1.19	.000	1.11	1.26
**Highest educational level**						
	No education ^rc^					
	Primary	-0.04	0.96	.249	0.89	1.03
	Secondary	0.15	1.17	.000	1.09	1.25
	Higher	0.22	1.25	.009	1.06	1.47
**Media exposure**						
	No ^rc^					
	Yes	0.19	1.20	.000	1.13	1.28
**Body Mass Index**						
	Underweight ^rc^					
	Normal	0.03	1.03	.327	0.97	1.09
	Overweight	0.14	1.15	.006	1.04	1.27
**Tetanus injection received**						
	None ^rc^					
	1	0.26	1.30	.000	1.16	1.45
	2	0.38	1.46	.000	1.34	1.59
	3+	0.55	1.73	.000	1.56	1.91
**Antenatal care visits**						
	No visits ^rc^					
	1–3 visits	0.32	1.37	.000	1.28	1.47
	4+ visits	0.77	2.16	.000	2.01	2.32
**Age at first birth**						
	≤18^rc^					
	19–23	0.01	1.01	.659	0.96	1.08
	24+	0.08	1.08	.060	0.10	1.17
**Wealth index**						
	Poor ^rc^					
	Middle	0.23	1.26	.000	1.17	1.36
	Rich	0.50	1.64	.000	1.51	1.79
**Religion**						
	Muslim ^rc^					
	Hindu	0.12	1.15	.000	1.07	1.23
	Others	-0.12	0.88	.008	0.81	0.97
**Birth order**						
	Five or more ^rc^					
	Four	0.13	1.14	.007	1.04	1.26
	Third	0.25	1.28	.000	1.17	1.40
	Second	0.36	1.43	.000	1.31	1.56
	One	0.42	1.51	.000	1.38	1.66

Key notes.

^B^ Estimated Coefficient

^rc^ Reference Category

^AOR^ Adjusted Odds Ratio

The women from urban areas were 1.19 times (AOR: 1.19, 95% CI: 1.11–1.26) more likely to avail home-based delivery by skilled birth attendants compared to those from rural areas. Women with secondary and higher level of education were 1.17 times (AOR: 1.17, 95% CI: 1.09–1.25) and 1.25 times (AOR: 1.25, 95% CI: 1.06–1.47) more likely to use skilled birth attendants during delivery compared to the woman with no education. Moreover, women exposed to media were 20% (AOR: 1.20, 95% CI: 1.13–1.18) more likely to be delivered by skilled birth attendants than women unexposed to media. The odds of having home-based delivery by skilled birth attendants are 51% higher among women during their first birth (AOR: 1.51, 95% CI: 1.38–1.66) compared to their fifth and later births. Women who are overweight had 1.15 times (AOR: 1.15, 95% CI: 1.04–1.27) higher odds of using skilled birth attendants than women in the underweight group. Similarly, the odds of having home-based skilled birth attended delivery among women in affluent households were 1.64 (AOR: 1.64, 95% CI: 1.51–1.79) times higher than women in poor households. Women who had received 3+ Tetanus injections were 73% (AOR: 1.73, 95% CI: 1.56–1.91) more likely of having skilled birth delivery than women who did not receive Tetanus injections at all. Furthermore, women who had 4+ antenatal care visits for their most recent delivery were 2.16 (AOR: 2.16, 95% CI: 2.01–2.32) times more likely to have home-based skilled birth attendance compared to those who had no antenatal care visits. Besides, Hindu women had 1.15 (AOR: 1.15, 95% CI: 1.07–1.23) times higher odds of using skilled birth attendance during delivery compared to Muslim women.

The Log-Binomial Logistic Regression, as shown in Table 4, also reveals a highly significant association between the utilization of home-based skilled birth attendance and the predictors such as type of place, educational level, religion, media exposure, body mass index, wealth index, tetanus injection receiving status, number of antenatal care visits and birth order.

**Table 4:** Results of Log-Binomial Logistic Regression (LBLR) model estimates showing adjusted odds ratios (AORs) (including 95% confidence intervals) and the determinants of home-based skilled birth attendance.

		B	AOR	P-value	95% Wald Confidence Interval	
					Lower	Upper
**Type of place**						
	Rural ^rc^					
	Urban	0.13	1.14	.000	1.08	1.20
**Highest educational level**						
	No education ^rc^					
	Primary	-0.03	0.98	.403	0.92	1.03
	Secondary	0.12	1.13	.000	1.07	1.18
	Higher	0.14	1.15	.009	1.04	1.28
**Media exposure**						
	No ^rc^					
	Yes	0.16	1.17	.000	1.11	1.23
**Body Mass Index**						
	Underweight ^rc^					
	Normal	0.02	1.02	.395	0.97	1.07
	Overweight	0.10	1.11	.006	1.03	1.19
**Tetanus injection received**						
	None ^rc^					
	1	0.22	1.25	.000	1.13	1.37
	2	0.32	1.38	.000	1.28	1.48
	3+	0.44	1.56	.000	1.43	1.69
**Antenatal care visits**						
	No visits ^rc^					
	1–3 visits	0.27	1.31	.000	1.24	1.38
	4+ visits	0.59	1.81	.000	1.71	1.92
**Age at first birth**						
	≤18 ^rc^					
	19–23	0.01	1.01	.753	0.96	1.06
	24+	0.06	1.06	.078	0.10	1.12
**Wealth index**						
	Poor ^rc^					
	Middle	0.18	1.20	.000	1.13	1.26
	Rich	0.34	1.41	.000	1.33	1.49
**Religion**						
	Muslim ^rc^					
	Hindu	0.12	1.12	.000	1.07	1.18
	Others	-0.06	0.94	.113	0.88	1.01
**Birth order**						
	Five or more ^rc^					
	Four	0.12	1.13	.004	1.04	1.22
	Third	0.21	1.23	.000	1.14	1.32
	Second	0.29	1.34	.000	1.25	1.44
	One	0.34	1.40	.000	1.30	1.51

Key note.

^B^ Estimated Coefficient

^rc^ Reference Category

^AOR^ Adjusted Odds Ratio

Urban women had 1.14 times (AOR: 1.14, 95% CI: 1.08–1.20) more likelihood to have home-based delivery by skilled birth attendants compared to rural women. The higher educated women had 15% (AOR: 1.15, 95% CI: 1.04–1.28) more likelihood to use skilled birth attendance during delivery than the women who had no formal education. Regarding media exposure, women exposed to media were 1.17 (AOR: 1.17, 95% CI: 1.11–1.23) times more likely to be delivered by skilled birth attendants than women unexposed to media. Also, women in the overweight group had 11% (AOR: 1.11, 95% CI: 1.03–1.19) higher likelihood of using skilled birth attendants than women in the underweight group. Similarly, the higher odds (41%) of having home-based skilled birth attended delivery among women were observed among women from affluent households (AOR: 1.41, 95% CI: 1.33–1.49) than women from poor households. Women who had received 3+ Tetanus injections were 56% (AOR: 1.56, 95% CI: 1.43–1.69) more likely of having skilled birth delivery at home than women who did not receive Tetanus injections at all. Similarly, women who had 4+ antenatal care visits for their most recent delivery were 1.81 (AOR: 1.81, 95% CI: 1.71–1.92) times more likely to have home-based skilled birth attendance compared to those who had no antenatal care visits. Hindu women had 1.12 (AOR: 1.12, 95% CI: 1.07–1.18) times higher odds of using skilled birth attendants during delivery compared to Muslim women. The odds of having home-based delivery by skilled birth attendants among the woman during their first birth were 1.40 times (AOR: 1.40, 95% CI: 1.30–1.51) higher compared to their fifth born.

### Goodness of fit test

Table 5 denotes that Log-Binomial Logistic Regression model is more favored than the Binomial Logistic Regression model based on the two goodness of fit measures: AIC and BIC. In addition, the table specifies that the assessed AIC and BIC are smaller for the Log- Binomial Logistic Regression model than the Binomial Logistic Regression model.

**Table 5:** Comparison table of two statistical model.

Model	Akaike’s Information Criterion (AIC)	Bayesian Information Criterion (BIC)
Binomial Logistic Regression Model	37666.30	37864.68
Log-Binomial Logistic Regression Model	18142.55	18340.94

## Discussion

The goal of this study was to investigate the disparities in Indian women’s use of competent delivery help during labor at home and to identify the factors associated with the use of skilled birth attendance during delivery at home. We concluded that Log-Binomial Logistic Regression (LBLR) is the best fit model for this study after assessing AIC and BIC. According to the findings of the LBLR investigation, women who live in urban areas, are well educated, and have regular media exposure are more likely to use a competent birth attendant even when they give birth at home. The study also revealed that women with overweight and from affluent households in India are more likely to use SBA at home during childbirth. Women who receive prenatal care during pregnancy and receive a tetanus shot and at their early pregnancies are more likely to request for SBA during childbirth at home.

Literature also suggests that urban women are more likely to have skilled birth attendance compared to the rural women [34, 35]. The healthcare system in rural India does not have adequately trained support staffs which makes it difficult for the rural people to access health care services when needed [36]. The relative unavailability of skilled birth attendance in rural area might result in lower incidence of giving birth in the presence of a skilled birth attendant at home by the rural Indian women. Studies also suggest that women with higher level of education are more likely to give birth in the presence of a skilled birth attendant [37, 38] which is reconfirmed in our study. Women with higher education are supposed to be more aware about the complicacies related to pregnancy and more capable to understand the role of skilled birth attendance in safe delivery. Therefore, it is likely that educated women, even while giving birth at home, will avail the service of a skilled birth attendant.

It is also found in our study that Hindu women are more likely to have home-based skilled birth attendance compared to Muslim women. In the literature, religion has been identified as a significant predictor of having skilled birth attendance [39, 40]. A study also found that Muslim women are usually less likely to use skilled birth attendance and safe deliveries compared to Hindu women [41]. It is difficult to explain the variation in the use of skilled birth attendance at home based on religion. Therefore, further research is warranted to explore how an individual’s belief system is associated with decision on availing professional health care services like skilled birth attendance.

Despite the fact that a large percentage of educated women from wealthy Hindu families are considering SBA during childbirth, the number of women who are unaware of the benefits of SBA even during home-based childbirth is not insignificant which needs immediate actions by the government of India.

Our research further suggests that media exposure is positively associated with home based skilled birth attendance. Prior works suggests the same that women who are more exposed to mass media are more likely to give birth in the presence of a skilled birth attendant [42–44]. Mass media helps people know about complicacies that may arise during giving birth and how a skilled birth attendant can be a life savior which probably led women with high media exposure to use skilled birth attendance at home as well.

This study found that overweighted women are more likely to seek help from skilled birth attendants while giving birth at home compared to women who are underweighted. Literature showing relationship between BMI and skilled birth attendance is extremely scarce. A study, conducted on women in Bangladesh, found that women whose weight is not normal are more likely to use skilled birth attendance compared to normal-weighted women [44]. High BMI is associated with a number of health risks such as diabetes, cardiovascular diseases, kidney diseases, stroke etc. [45]. Women, due to being overweighted and having a number of associated health complicacies, are more likely to be under supervision of a professional medical practitioner and therefore, are more likely to be aware about the complicacies that may occur during pregnancy and child-birth. They are supposed to receive suggestions to seek professional help even if they give birth at home which may lead to higher likelihood of availing skilled birth attendance at home by them.

Our research found that women from affluent households in India are more likely to ask for help from skilled birth attendants while giving birth at home compared to women from poorer households. Literature, however, shows mixed results on the association between wealth index and use of skilled birth attendance. For example, a study found that women who belong to rich household have higher odds of having skilled birth attendance than the women who are from poor families which is in line with our findings [46]. Contrary to that, another study found that poorer women are more likely to use skilled birth attendance at home compared to women from rich households [47]. Therefore, we don’t have a plausible explanation on why women from affluent households are more likely to use skilled birth attendance at home and through this study, we call for further research on this.

Generally, low-income families choose to give birth at home with the help of unskilled SBAs due to the low cost and the benefit of payment negotiation [48]. This behavior can be reduced, especially for the poor, by lowering out-of-pocket costs for institutional delivery [49]. Effort should be made to improve the training of these birth attendants so that they can assist with childbirth at home and make referrals to the nearest healthcare institution when necessary [50].

Our study also found that women who received Tetanus injections were more likely to seek assistance from skilled birth attendants at home compared to women who did not receive the Tetanus shots at all. Research on the relationship between receiving TT injections and skilled birth attendance is extremely inadequate. A study found that TT injection can reduce neonatal mortality [51]. Tetanus is a disease which is still prevalent across the world and can lead to fatal outcomes [52, 53]. A mother who is conscious about neonatal mortality and other fatalities that are associated with tetanus and therefore takes tetanus shots has high level of awareness and is supposed to be careful about safe delivery as well. Therefore, a mother who receives tetanus injection should be more likely to use skilled birth attendance while giving birth at home. Our study also found that people who are more likely to receive antenatal care (ANC) services are more likely to have home based skilled birth attendance which is in line with the findings of the literature showing positive relationship between number of visits for ANC services and availing skilled birth attendance [54, 55]. People who avail ANC services and skilled birth attendance belong to the same cohort almost [56, 57]. Therefore, it is likely that women who avail ANC services will also go for skilled birth attendance. Efforts should be made to make SBA conveniently accessible along with prenatal and postnatal care services during the first 24 hours of birth to minimize the high maternal mortality rate in the low-income countries like India [10, 58].

Findings from our study also suggest that birth order has a significant relationship with availing skilled birth attendance while giving birth at home. Women in their early pregnancies are more likely to use home-based skilled birth attendance compared to later pregnancies. Similar studies also found that with later pregnancies, women are less likely to ask for skilled birth assistance [44, 59]. During the first couple of pregnancies women may feel insecure as they don’t have prior experience of child-birth. They may tend to be extra-cautious for safe delivery. Once they get accustomed to the procedures related to child-birth, they may feel that they don’t need expert handling of the pregnancies which may lead to lower usage of skilled birth attendance at home by the Indian women.

## Strength and limitation

This study is one of its kind as it explored factors that influence availing the assistance of a skilled birth attendant while giving birth at home unlike other studies that explored factors related to availing institutional skilled birth attendance. The novelty of our studies lies in identifying some of the factors that have not been investigated thoroughly by the prior studies such as BMI, status of tetanus injection reception, birth order using a nationally representative data. However, there are some limitations associated with this study. The DHS data used in this study encompassed a wider range of locations and time points, which introduced selection bias. Furthermore, each variable was divided into two categories before the OR was calculated. In addition, we were unable to incorporate all the potential risk factors such as cost of delivery, geographical locations and the availability of other medical schemes.

Further research should investigate those newly identified factors. Further research should also attempt to explain how belief system, BMI, status of Tetanus injection reception and birth order affect decision on availing skilled birth attendance at home. The study calls for public campaign and social mobilization to raise awareness among the general populace so that they seek skilled birth attendance in cases where women are giving birth at home as well. This study also urges for greater access to the healthcare services so that home- based SBA takes place to a larger extent. Appropriate policy interventions by the governments, therefore, has a key role to play here.

## Conclusions

This study intends to explore the factors affecting home-based skilled birth attendance among the Indian Women. Data was taken from the Indian Demographic and Health Survey 2015–16 also termed as the National Family Health Survey-4 (NFHS-4). Analysis of the data suggests that a number of factors such as place of living, level of education, religion, media exposure, body mass index, possession of wealth, status of tetanus injection reception, number of antenatal care visits and birth order affect the availing of skilled birth attendance by the Indian women while giving birth at home. The important strategy for India, as demonstrated by earlier studies is to increase the rate and the availability of skilled birth attendants during childbirth at home in order to lessen the burden of maternal and child mortality and to achieve maternal and child health-related goals [60–63].

## List of abbreviations

SBA: Skilled Birth Attendants
AOR: Adjusted Odds Ratio
CI: Confidence Interval
rc: reference category
ANC: Antenatal Care
AIC: Akaike information criterion
BIC: Bayesian information criterion
BLR: Binary Logistic Regression
LBLR: Log-Binary Logistic Regression
